# Alteration of Bile Acids and Omega-6 PUFAs Are Correlated With the Progression and Prognosis of Drug-Induced Liver Injury

**DOI:** 10.3389/fimmu.2022.772368

**Published:** 2022-04-12

**Authors:** Shuang Zhao, Haoshuang Fu, Tianhui Zhou, Minghao Cai, Yan Huang, Qinyi Gan, Chenxi Zhang, Cong Qian, Jiexiao Wang, Zhenglan Zhang, Xiaolin Wang, Xiaogang Xiang, Qing Xie

**Affiliations:** Department of Infectious Diseases, Ruijin Hospital, Shanghai Jiao Tong University School of Medicine, Shanghai, China

**Keywords:** DILI, chronicity, BAs, PUFAs, gut microbiota

## Abstract

**Background & Aims:**

Drug-induced liver injury (DILI) is one of the leading causes of liver failure with some of the patients progressed to chronic DILI. The mechanisms underlying the severity and chronicity of DILI are poorly elucidated and the biomarkers are limited. Metabolites and gut microbiota played a crucial role in the development of various liver diseases. Herein, a systematic analysis of serum metabolites and gut microbiota was performed in DILI patients, aiming to identify metabolites correlated with the progression and clinical prognosis of DILI.

**Methods:**

Various serum metabolites were quantitated using a metabolite array technology in this prospective study. Gut microbiome compositions and the expression profiles of liver genes were determined in patients with DILI and healthy controls.

**Results:**

Metabolomic analysis revealed that bile acids (BAs) and polyunsaturated fatty acids (PUFAs) were closely related to DILI severity and chronicity respectively. The ratios of serum primary/secondary BAs and omega-6/omega-3 PUFAs were elevated in DILI patients. A model established by adrenic acid (AdA) and aspartic acid (Asp) exerts good performance for predicting the chronicity of DLIL. Hepatic transcriptome revealed enhanced expression of PUFA peroxidation and supressed expression of BA synthesis related genes in DILI patients. In addition, Lactic acid bacteria and BA converting bacteria were increased in gut of DILI patients. Besides, elevated serum malondialdehyde (MDA) and fibroblast growth factor 19 (FGF19) was observed in DILI patients.

**Conclusion:**

BAs and PUFAs could be potent markers for the severity and chronicity of DILI respectively. The panel of AdA and Asp could be ideal predictive model for the risk of chronicity at the acute stage of DILI. Gut microbiota might act as a negative feedback mechanism to maintain the homeostasis of BAs and PUFAs *via* FGF19 signalling and PUFA saturation, respectively. Our study revealed novel biomarkers for severe and chronic DILI and provided new therapeutic targets for DILI.

## Introduction

Drug-induced liver injury (DILI) is an adverse liver reaction to numerous drugs, including drugs, toxins, and herbal medicines with an annual incidence of 13-24 per 100,000 person-year worldwide. Severe DILI can lead to liver failure, the need for liver transplantation, or death (1–3). On the other hand, about 6-39% of patients progressed to chronic DILI (4–9) which could lead to repeated liver inflammation and even cirrhosis and remains a notable challenge in clinical practice. Therefore, early identification of chronic DILI is crucial for preventing the associated negative consequences. Up till now, there have been no widely accepted biomarkers for evaluating the progression and prognosis of DILI, thus identification of non-invasive biomarkers for the severity and chronicity of DILI is urgently needed.

Since the liver is a major metabolic hub in the human body, probing the metabolic signature of DILI holds promise for the discovery of new biomarkers for disease severity, prognosis, and even therapeutic targets. For example, accumulating evidence highlighted the promise of BAs as biomarkers for the diagnosis and prognosis of severe DILI (10). Recently, Jia et al. found that serum taurocholic acid (TCA) could serve as a potential biomarker for chronic DILI (11). Dyslipidemia was found as a risk factor for the severity of DILI and favored a chronic outcome (12, 13). Moreover, lipid peroxidation was found to aggravate anti-tuberculosis DILI (14). These studies implied that abnormal metabolites might correlate with DILI progression. Microbiota aberrations were recently found to be associated with many hepatic diseases (15), such as non-alcoholic fatty liver diseases (16), liver cirrhosis (17), chronic hepatitis B (18) and liver cancer (19). However, few studies focused on gut microbiota in DILI patients. Thus, we investigated 16S RNA of faeces to elucidate the compositional and functional characteristics of the gut microbiota in the acute stage and to understand their influence on metabolism and disease progression.

The emerging automated high-throughput metabolite array technology (20) allowed the metabolic alterations in DILI to be detected comprehensively and quantitatively. In this prospective study, we analyzed and compared various metabolic components between healthy controls (HCs) and DILI patients. To understand the possible mechanisms by which metabolites participate in DILI progression, gut microbiota community and hepatic metabolism related gene expression were also detected. The present results may shed new light into the mechanisms underlying DILI progression and provide potential therapeutic targets for the treatment of DILI.

## Materials and Methods

### Ethics Statement

The study was approved by the Ethics Committee of Shanghai Ruijin Hospital, School of Medicine, Shanghai Jiao Tong University in accordance with the Helsinki Declaration. Written informed consents were provided for all patients prior to participation.

### Subjects

**Figure 1:**
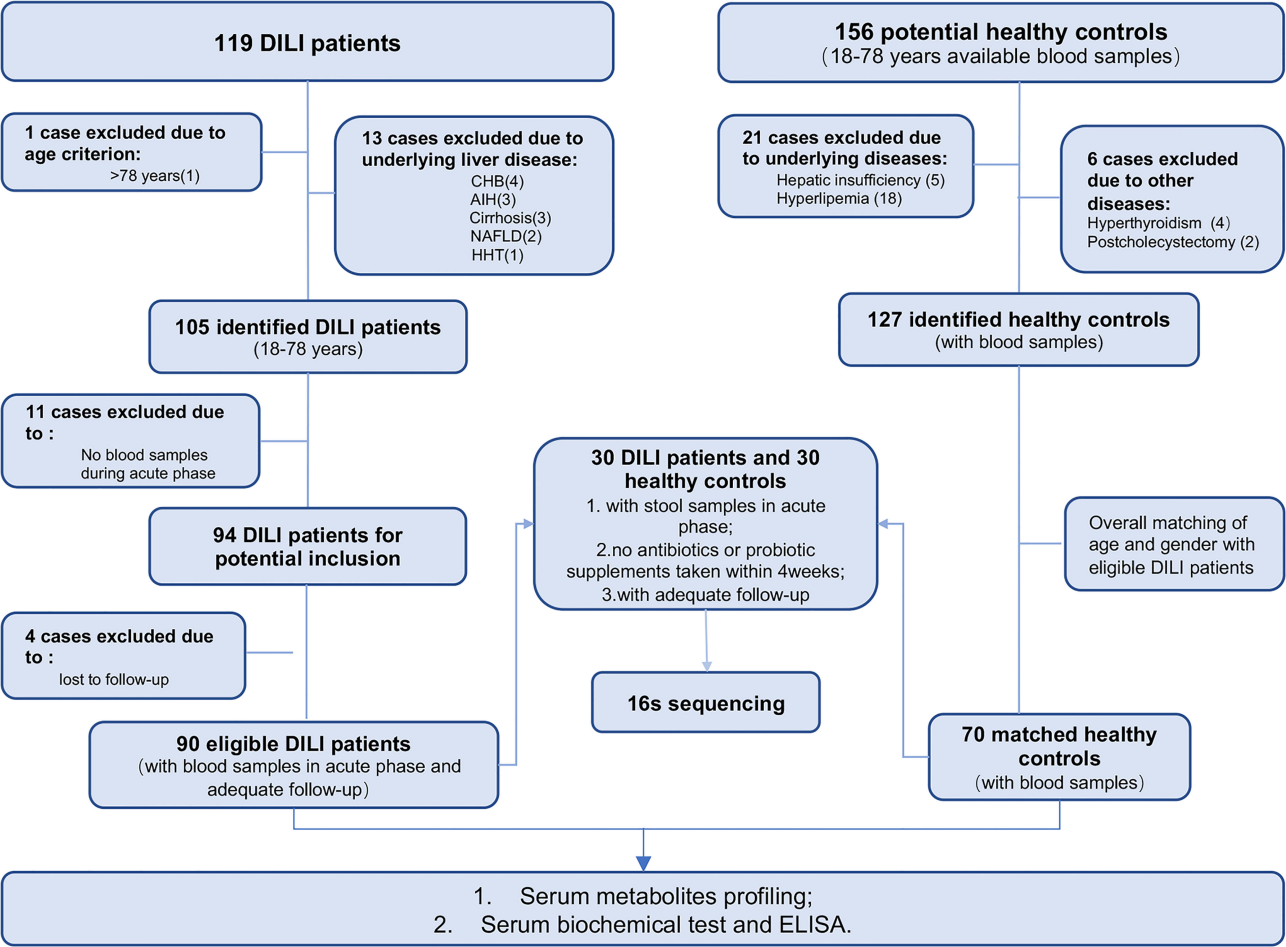
Enrollment of DILI patients and healthy control subjects. CHB, chronic hepatitis B; AIH, autoimmune hepatitis; NAFLD, nonalcoholic fatty liver disease; HHT, hereditary hemorrhagic telangiectasia; MDA, malondialdehyde; GSH-Px, glutathione peroxidase; FGF19, fibroblast growth factor 19. (Created with BioRender.com.).

One hundred and nineteen DILI patients and 156 healthy controls (HCs) were recruited in this study. A total of 90 hospitalized DILI patients and 70 HCs were included in the final analysis (recruitment flowchart provided in **Figure 1**).

Criteria for DILI patients enrollment included (i) diagnosed as DILI according to the Roussel Uclaf Causality Assessment Method (Rucam) score ≥6, (ii) patients aged from 18 to 78 years, and (iii) available blood samples from the acute phase (within 10 days of obtaining the peak value of transaminase or bilirubin). Exclusion criteria for patients were as follows: (i) other pre-existing liver disease or systemic diseases affecting the liver;(ii) alcohol consumption > 40g/day; (iii) no blood samples during acute phase and;(iv) patients lost to follow-up. For fecal 16S rRNA sequencing, individuals currently taking antibiotics or probiotic supplements within one month prior to sample collection were also excluded.

DILI chronicity was defined as persistent serum levels of aspartate aminotransferase (AST), alanine aminotransferase (ALT), or alkaline phosphatase (ALP) (abnormal liver biochemistry) above baseline after 6 months from DILI onset, regardless of the types of liver injury (21, 22). The severity of DILI was categorized as one of the four degrees (Grade 1-mild, Grade 2-moderate, Grade 3-severe, Grade 4-liver failure) according to the previously published guideline for DILI in our study (22).

Age and gender matched healthy individuals were enrolled as controls (**Supplementary Material**). Peripheral blood samples (anticoagulated by EDTA) of HCs and DILI patients were obtained from Ruijin Hospital. All patients were followed up for at least 6 months. Relevant clinical data were abstracted from the medical record.

### Metabolite Array Technology

Serum from DILI patients and HCs were collected and frozen at -80°C for the subsequent metabolism measurement. Comprehensive quantitation of serum metabolism components including fatty acids, amino acids, organic acids, carbohydrates, and BAs were performed with an ultra-performance liquid chromatography coupled to tandem mass spectrometry (UPLC-MS/MS) system (ACQUITY UPLC-Xevo TQ-S, Waters Corp., Milford, MA, USA) according to recently published methods (20). All of the standards of targeted metabolites were obtained from Sigma-Aldrich (St. Louis, MO, USA), Steraloids Inc. (Newport, RI, USA) and TRC Chemicals (Toronto, ON, Canada). Metabolite identification and quantification were achieved using the Targeted Metabolome Batch Quantification (TMBQ) software (v1.0, HMI, Shenzhen, Guangdong, China).

### 16S rRNA Sequencing

Fecal samples were collected in the morning and then stored at -80 °C prior to processing. Total fecal DNA was extracted using a QIAamp DNA Stool Mini Kit (Qiagen, Hilden, Germany). Sequencing libraries were generated using TruSeq^®^ DNA PCR-Free Sample Preparation Kit (Illumina, USA) following the manufacturer’s recommendations and quality was controlled by TapStation. The 16S recombinant DNA sequencing data were processed by the ucluster method of Quantitative Insights into Microbial Ecology (QIIME) (V1.9.1), then the effective Tags were obtained. Operational taxonomic unit (OTU) Production Sequences analysis was performed by Uparse software (Uparse v7.0.1001). Subsequent analysis of alpha diversity and beta diversity were all performed based on this output normalized data by QIIME (Version 1.7.0) and QIIME (Version 1.9.1) respectively.

### PCR-Array

Human liver tissue from 4 DILI patients and 3 healthy donors were homogenized and total RNA isolated using the trizol reagent. Sequencing libraries were generated using NEBNext^®^ Ultra™ RNA Library Prep Kit for Illumina^®^ (#E7530L, NEB, USA) following the manufacturer’s recommendations and index codes were added to attribute sequences to each sample. RNA concentration of library was measured using Qubit^®^ RNA Assay Kit in Qubit^®^ 3.0 to preliminary quantify and then dilute to 1ng/μl. Insert size was assessed using the Agilent Bioanalyzer 2100 system (Agilent Technologies, CA, USA), and qualified insert size was accurate quantification using StepOnePlus™ Real-Time PCR System (Library valid concentration>10 nM). The clustering of the index-coded samples was performed on a cBot cluster generation system using HiSeq PE Cluster Kit v4-cBot-HS (Illumina) according to the manufacturer’s instructions. After cluster generation, the libraries were sequenced on an Illumina platform and 150 bp paired-end reads were generated. The gene expression values in pathways were calculated by HTSeq v0.6.0 and FPKM.

### Biochemical Test and ELISA

Serum of DILI and healthy controls were collected and frozen at -80℃ for the subsequent biochemical or ELISA measurement. The oxidative stress and antioxidant markers MDA and Glutathione peroxidase (GSH-Px) activity were measured following the manufacturer’s protocol (Nanjing Jiancheng Bioengineering Institute, Nanjing, China). The absorbance was estimated by a microplate reader (BioteK, USA). Serum FGF19 measurement was performed using an ELISA kit from R&D Systems following the manufacturer’s protocol.

### Statistical Analysis

Categorical variables were expressed as counts or percentages, continuous variables were presented as mean ± SD or median and range. For normally distributed data, an unpaired student’s t-test was used for comparison between two groups, and one-way ANOVA was used for multiple comparisons among different groups. For non-normally distributed data, a Mann–Whitney U-test was used for comparison between two groups, and a non-parametric Kruskal–Wallis test was used for multiple comparisons among different groups. Chi-square analysis was applied for categorical data. For correlation analysis, we use the Pearson correlation test. Univariate and multiple logistic regression analysis was performed to establish the predictive model. Data were analyzed using SPSS 22.0 software (SPSS Inc, Chicago, IL). Software packages in R studio (R version 4.1.0, the R Foundation for Statistical Computing) were used for other statistical analyses including principal component analysis (PCA), random forest (RF), Volcano plot, and to plot ROC, nomogram, calibration plot and decision curve analysis plot. For all tests, a two-tailed p < 0.05 was considered statistically significant.

## Results

### Clinical Characteristics of the Recruited DILI Patients and Healthy Controls

In our study, 90 DILI patients and 70 HCs were included. DILI patients were divided into two groups according to outcome: the recovered DILI (DILI.rec) and chronic DILI (DILI.chr) group. According to the criteria of DILI severity (22), patients were classified as mild (Grade 1), moderate (Grade 2), severe (Grade 3), and liver failure (Grade 4). Moreover, we defined Grade 1 and Grade 2 as non-critical group (DILI.a), and Grade 3 and Grade 4 as critical group (DILI.b). The comparisons of characteristics between the different groups were shown in **Table 1**. The flowchart illustrated the recruitment of patients with DILI and HCs based on the exclusion and inclusion criteria (**Figure 1**). Detailed drug information was summarized in **STable 1**. Most of the DILI cases were female (63.3%) and the majority were due to taking herbs (66.7%).

**Table 1:** Demographics and Baseline Characteristics of Healthy Controls and DILI Patients.

Parameters	HC n = 70	Overall DILI n = 90	DILI.rec n =65	DILI.chr n =25	DILI.a n=54	DILI.b n=36	*P* value	*P* value^*^	*P* value^#^
Age	54 (49–57)	58 (44–66)	59 (43-66)	58 (51-71)	58 (46-66)	61 (41-68)	0.0924	0.2384	0.9632
Sex (*M/F)*	27/43	33/57	24/42	10/15	16/38	17/19	0.8697	0.8103	0.1188
ALT (*IU/L*)	20.2 ± 11.1	489 ± 554.8	575.2 ± 589.8	366.3 ± 451.8	413.2 ± 573.2	602.8 ± 512.9	<0.0001	0.1422	0.1128
AST (*IU/L*)	21.1 ± 6.1	335.9 ± 366.5	349.9 ± 309.7	386.2 ± 519.3	227.3 ± 256.7	498.7 ± 43.1	<0.0001	0.9940	0.0004
ALP (*IU/L*)	65.9 ± 17.8	209.7 ± 149.4	228.9 ± 165.5	173.8 ± 83.68	185.7 ± 119.7	245.8 ± 181.3	<0.0001	0.1312	0.0613
GGT (*IU/L*)	20.8 ± 13.8	278.9 ± 257.4	303.6 ± 280.3	237.6 ± 176.6	235.9 ± 195.0	335.9 ± 323.9	<0.0001	0.3042	0.0710
Tbil (*μmol/L*)	13.2 ± 4.6	114.4 ± 113.4	106.7 ± 112.1	167.1 ± 114.2	41.57 ± 33.01	223.5 ± 103.1	<0.0001	0.0356	<0.0001
Dbil (*μmol/*)	2.3 ± 0.8	57.7 ± 58.7	51.43 ± 58.1	83.3 ± 54.4	18.16 ± 19.55	110.3 ± 52.7	<0.0001	0.0329	<0.0001
TBA (*μmol/*)	3.4 ± 3.1	98.8 ± 90.8	91.5 ± 96.7	120.1 ± 68.2	39.07 ± 7.976	33.9 ± 11.2	<0.0001	0.2041	0.0127
INR (*mg/dL*)	NT	1.3 ± 1.5	1.3 ± 1.7	1.2 ± 0.5	1.334 ± 1.831	1.2 ± 0.4	NA	0.8347	0.7335
AFP (*ng/mL*)	NT	20.3 ± 79.6	20.7 ± 90.0	18.8 ± 29.3	7.193 ± 8.364	37.8 ± 121.5	NA	0.9280	0.0814

Data are expressed as Mean ± SD or median and interquartile (25th and 75th).

ALP, alkaline phosphatase; ALT, alanine aminotransferase; AST, aspartate aminotransferase; DBil, conjugated bilirubin; DILI, drug-induced liver injury; DILI.rec, DILI recoverd within 6 months; DILI.chr, chronic DILI; GGT, gamma-glutamyl transferase; HC, healthy control; Ig, immunoglobulin; INR, international normalized ratio; NA, not available for statistically significance analysis; NT, not tested; TBA, total bile acids; TBil, total bilirubin.

P value stands for the overall comparison of HCs and overall DILI.

^*^P value stands for the overall comparison of DILI.rec and DILI.chr

^#^P value stands for the overall comparison of DILI.a and DILI.b

### Differences in Metabolites Between DILI and HCs Were Identified by Global Metabolic Analysis

**Figure 2:**
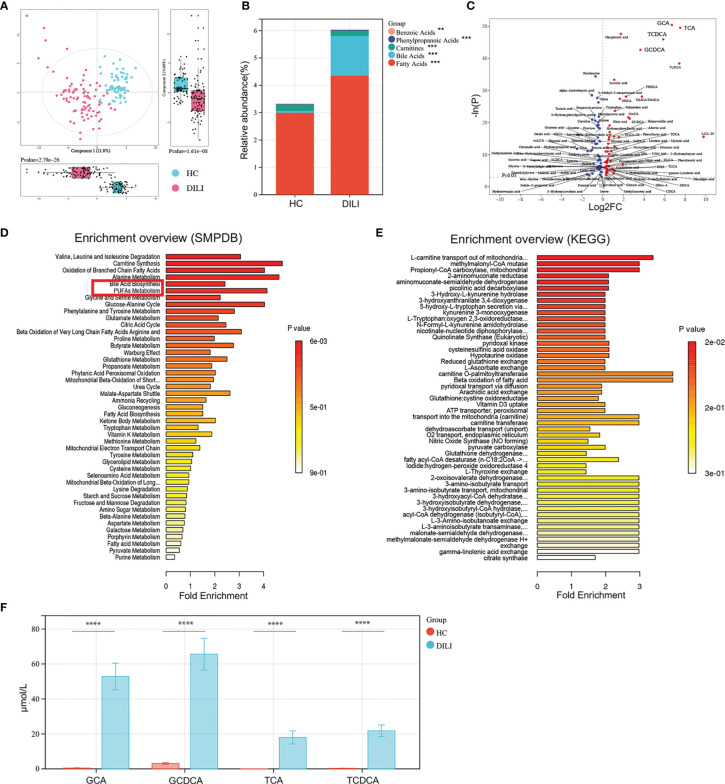
Examination of the metabolic profiles of HCs and DILI patients. **(A)** The PCA plot showed a clear discrimination of serum metabolic profiles between DILI patients and HCs. **(B)** The relative abundance of mainly differential metabolite classes in HC and DILI group. **(C)** Volcano plot of the most significant metabolite changes comparing HCs with DILI patients. **(D, E)** Pathway analysis used to discover the differences of metabolic characteristics between DILI and HCs (based on KEGG or SMPDB). **(F)** Boxplot of the four most significant metabolites in the analysis of volcano plot comparing HCs and DILI patients. **p < 0.01; ***p < 0.001; ****p < 0.0001. PCA, principal component analysis; PUFA, polyunsaturated fatty acid; SMPDB, human metabolome database; KEGG, kyoto encyclopedia of genes and genomes; GCA, glycocholic acid; GCDCA, glycochenodeoxycholic acid; TCA, taurocholic acid; TCDCA, taurochenodeoxycholic acid.

To understand the metabolites that are involved in the progression of DILI, a high-throughput metabolite array was performed using serum from HCs and DILI patients. As illustrated by PCA score plot, the discrimination trends among samples from HCs and DILI patients revealed significant systematic metabolic differences (**Figure 2A**). The stacked bar chart showed that BAs and fatty acids were the main altered metabolites in DILI patients (**Figure 2B**).

Furthermore, the volcano plot showed that serum levels of glycochenodeoxycholic acid (GCDCA), glycocholic acid (GCA), taurochenodeoxycholic acid (TCDCA), and taurocholic acid (TCA) were significantly higher in DILI patients compared to HCs (**Figures 2C, F** **)**. Pathway analysis based on the Small Molecule Pathway Database (SMPDB) revealed that BA biosynthesis, PUFA metabolism and oxidation of branched chain fatty acids pathways were upregulated in DILI compared with HCs (**Figure 2D**). Pathway analysis based on the Kyoto Encyclopedia of Genes and Genomes (KEGG) database showed that L-carnitine transport out of mitochondria *via* diffusion, methylmalonyl-CoA mutase and propionyl-CoA carboxylase were upregulated about 3 folds in DILI patients and these pathways were all enriched by GCA, GCDCA, TCA and TCDCA (**Figure 2E**).

### Altered BAs and PUFAs Were Correlated With the Severity of DILI

**Figure 3:**
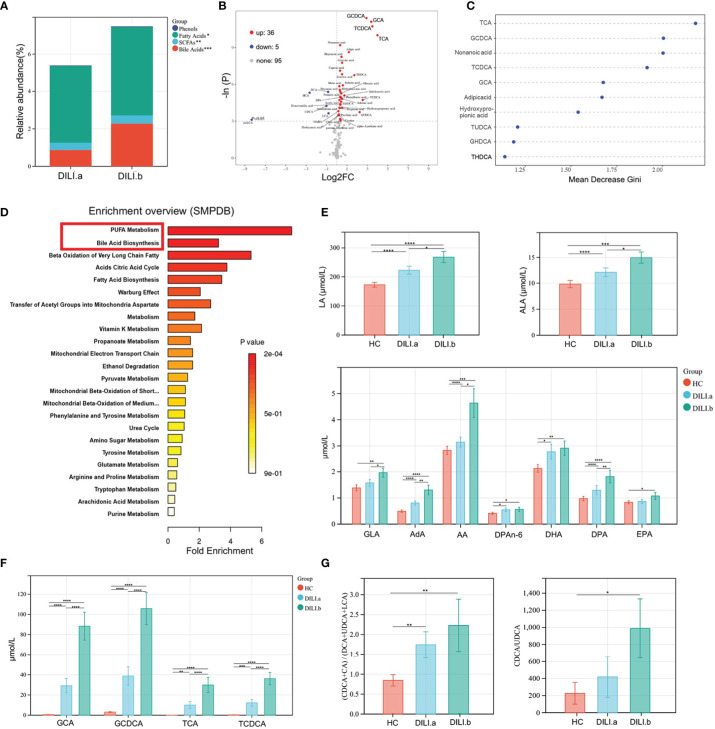
Examination of the metabolic profiles of DILI with different severity. DILI patients were categorized as mild (Grade 1), moderate (Grade 2), severe (Grade 3) and liver failure (Grade 4) groups. Grade 1 and Grade 2 were defined as DILI.a group, and Grade 3 and Grade 4 were defined as DILI.b group. **(A)** The relative abundance of differential metabolite classes between different groups of DILI. **(B)** Volcano plot of the most significant metabolite changes comparing DILI.a with DILI.b group. **(C)** Importance scores of top 10 important differential metabolites by RF between DILI.a and DILI.b. **(D)** A pathway analysis used to discover the different metabolic characteristics between DILI.a and DILI.b (based on SMPDB). **(E, F)** Boxplot of main differential bile acid and PUFAs. **(G)** Ratios of (CDCA+CA)/(DCA+UDCA+LCA) and CDCA/UDCA in HC, DILI.a and DILI.b groups. *p < 0.05; **p < 0.01; ***p < 0.001; ****p < 0.0001. RF, random forest; SCFA, short chain fatty acids; ALA, α-linolenic acid; LA, linoleic acid; AA, arachidonic acid; DPA, docosapentaenoic acid; GLA, gamma-linolenic acid; AdA, adrenic acid; CDCA, chenodeoxycholic Acid; CA, cholic acid; DCA, deoxycholic acid; UDCA, ursodeoxycholic acid; LCA, lithocholic acid.

Given that severe liver injury in DILI would progress into acute liver failure resulting in death or liver transplantation, we further divided DILI patients into DILI.a and DILI.b as mentioned earlier and explored the alteration of metabolic signature between the two groups. As illustrated by stacked bar chart in **Figure 3A**: BAs, short chain fatty acids (SCFAs) and fatty acids were the main altered metabolites between DILI.a and DILI.b group (**Figure 3A**). The volcano plot showed that GCDCA, GCA, TCDCA and TCA were the most significantly increased metabolites in DILI.b compared with DILI.a group (**Figure 3B**). Similar results were shown by random forest analysis (**Figure 3C**). SMPDB pathway analysis revealed a significant alteration in PUFA metabolism and BA biosynthesis pathway **(** **Figure 3D**). KEGG pathway analysis showed that L-carnitine transport out of mitochondria *via* diffusion, methylmalonyl-CoA mutase and propionyl-CoA carboxylase were upregulated nearly 4 folds in DILI.b compared with DILI.a group, and these pathways were enriched by GCA, GCDCA, TCA and TCDCA (**SFigure 1A**). Box plot showed that serum GCA, GCDCA, TCA, TCDCA and PUFA metabolites increased along with the severity of DILI (**Figures 3E, F** **)**. Further analysis revealed an imbalance in the ratio between primary BAs and secondary BAs. The ratios of (CDCA+CA)/(DCA+UDCA+LCA) and CDCA/UDCA, which represent the ratio of primary BAs and secondary BAs, were increased in both DILI.a and DILI.b groups (**Figure 3G**).

### PUFAs Were Most Correlated With the Chronicity of DILI

**Figure 4:**
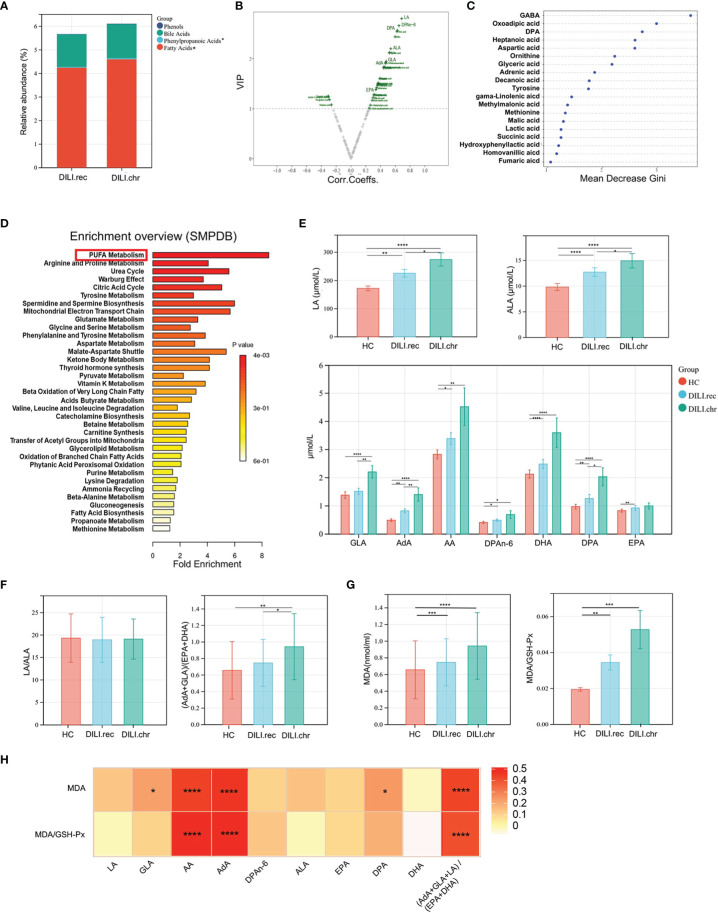
PUFAs were correlated with the chronicity of DILI. **(A)** The relative abundance of altered metabolite classes in recovered DILI (DILI.rec) and chronic DILI (DILI.chr) group. **(B)** Volcono plot of the most significant metabolite changes comparing DILI.rec with DILI.chr group. **(C)** Importance scores of top 20 important differential metabolites by RF between DILI.chr and DILI.rec. **(D)** A pathway analysis used to discover the different metabolic characteristics between DILI.rec and DILI.chr (based on SMPDB). **(E)** Boxplot of omega-6 PUFAs in HCs, DILI.rec and DILI.chr groups. **(F)** The ratios of LA/ALA and (AdA+GLA)/(EPA+DHA) between HC, DILI.rec and DILI.chr groups. **(G)** Serum levels of MDA and MDA/GSH-Px in HC, DILI.rec and DILI.chr groups. **(H)** Correlations of PUFAs with MDA and MDA/GSH-Px. *p < 0.05; **p < 0.01; ***p < 0.001; ****p < 0.0001. EPA, eicosapentaenoic acid; DHA, docosahexaenoic acid.

Chronic DILI manifests as persistent or repeated inflammation or diminishing bile ducts, which would progress into cirrhosis and even lead to liver transplantation, thus biomarkers for early prediction of chronic DILI were urgently needed. Patients were divided into recovered (DILI.rec) and chronic (DILI.chr) groups as we previously mentioned and metabolites were analyzed and compared between the recovered and chronic groups. As illustrated by stacked bar chart in **Figure 4A**, serum levels of fatty acids were significantly altered between DILI.rec and DILI.chr group. PUFA metabolites such as α-linolenic acid (ALA), γ-linolenic acid (GLA), linoleic acid (LA), adrenic acid (AdA), docosapentaenoic acid (DPA) and DPAn-6 were found as the major altered metabolites by volcano plot and random forest (**Figures 4B, C** **)**. Pathway analysis (SMPDB) revealed that the PUFA metabolism pathway plays an important role in DILI chronicity, which was mainly enriched by AdA, GLA and DPA (**Figure 4D**). The secondly upregulated pathway was arginine and proline metabolism, which was mainly enriched by fumaric acid, aspartic acid (Asp), ornithine and succinic acid (**Figure 4D**). Analysis based on the KEGG database showed upregulation of PUFA metabolism associated pathways such as carnitine O-palmitoyltransferase, beta oxidation of fatty acid, fatty acid transport *via* diffusion, transport into the mitochondria (carnitine), carnitine transferase, fatty-acid-CoA ligase, etc. (**SFigure 1B**). These pathways were enriched mainly by GLA and AdA. Further comparison confirmed that LA, GLA, AdA and Docosahexaenoic acid (DHA) differed significantly (p<0.05) between DILI.rec and DILI.chr group (**Figure 4E** and **SFigure 1C**).

Generally, PUFAs include omega-6 and omega-3 PUFAs. Omega-3 PUFAs mainly include ALA, eicosapentaenoic acid (EPA) and DHA which exert anti-inflammatory function, while omega-6 PUFAs mainly include LA, GLA, arachidonic acid (AA), AdA and DPAn-6, which are pro-inflammatory (23, 24). The level of omega-6 and omega-3 components of PUFAs were compared between DILI.rec and DILI.chr groups. There was no significant difference in the ratio of LA/ALA, while the ratio of (AdA+GLA+LA)/(EPA+DHA) was significantly increased (DILI.chr vs DILI.rec group) (**Figure 4F**). Malondialdehyde (MDA), a metabolic end product of PUFA which could be peroxidized by oxidative stress in DILI (25), has been widely used as a marker for lipid peroxidation (26). GSH-Px is the major antioxidant component in protecting the cells against increased reactive oxygen species (ROS) (27). Compared to HCs, serum level of MDA or MDA/GSH-Px in DILI patients was upregulated, a little higher in the DILI.chr group compared with the DILI.rec group (**Figure 4G**). And the level of AA, AdA or the ratio of (AdA+GLA+LA)/(EPA+DHA) was positively correlated with MDA and MDA/GSH-PX (**Figure 4H**).

### Establishment of a Predictive Model Based on Serum Metabolites

**Figure 5:**
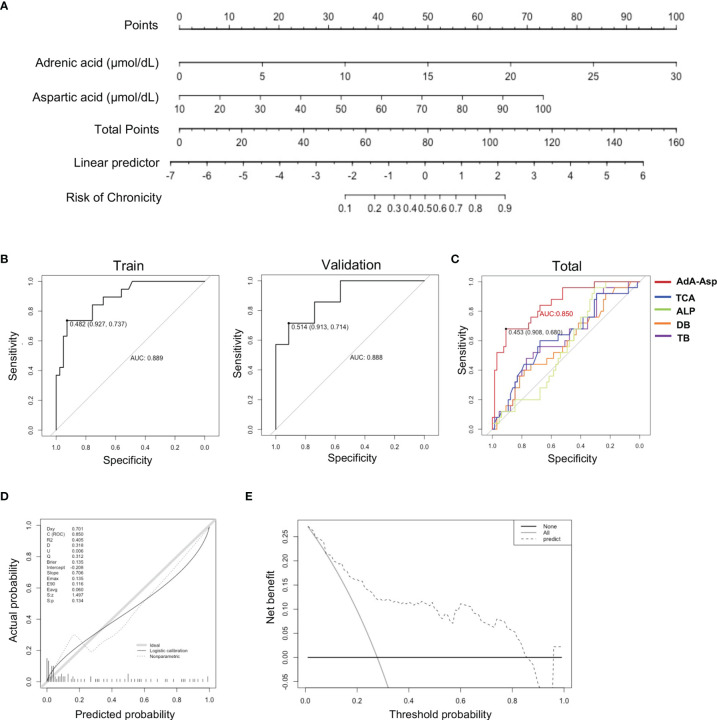
Predictive model establishment based on serum metabolites. **(A)** Nomogram based on serum metabolites for the prediction of DILI chronicity. (To use the nomogram, an individual patient’s value is located on each variable axis, and a line is drawn upward to determine the number of points received for each variable value. The sum of these numbers is located on the Total Points axis, and a line is drawn downward to the survival axes to determine the risk of chronicity in DILI.) **(B)** Plots of ROC results from the discovery set and the validation set.for distinguishing DILI.chr fromDILI.rec. The ROC curves were created by plotting the sensitivity (i.e., true positive rate) against 1-specificity (i.e., false positive rate). The line in each plot represents the area under the curve (AUC). **(C)** Plots of ROC results for distinguishing DILI.chr from the DILI.rec with ‘AdA-Asp model’ (Adrenic acid and Aspartic acid), TCA, ALP, DB and TB respectively. **(D)** Calibration plot to assess the accuracy of the model in the validation set. **(E)** Decision curve analysis plot to assess the clinical utility of the ‘AdA-Asp model’ (Decision curve analysis was based on a continuum of potential thresholds for chronicity risk (x axis) and the net benefit of using the model to risk stratify patients (y axis) relative to assuming that no patient will progress to chronic DILI. The thick black line represents the net benefit of assuming that none DILI patients would progress to chronic DILI; the thin grey line shows the net benefit of assuming that all would progress to chronic DILI. The black dotted line illustrates the net benefit of predicting the risk of DILI using the AdA-Asp model.). ROC, receiver operating characteristic; AUC, area under the curve; ALP, alkaline phosphatase; DB, direct bilirubin; TB, total bilirubin; Asp, aspartic acid.

It is promising to develop a model for predicting those who would convert into chronic DILI *via* these metabolites altered significantly. Random Forest, which copes well with high dimensional data, was used to select metabolites to discriminate chronic (DILI.chr) from recovered (DILI.rec) (GINI>1). The selected metabolites together with direct bilirubin (DB) and total bilirubin (TB) (t test p < 0.05) were determined as candidates for the predictive model. We then randomly divided the DILI patients into a training set (n=60) and a validation set (n=30) with a matched number of recovered and chronic patients. The univariate logistic regression performed in the training set identified DPA, aspartic acid, adrenic acid, tyrosine, GLA, fumaric acid, tyrosine, succinic acid and lactic acid as independent factors that predicted the risk of chronicity in DILI (**STable 2**). Subsequently, a binary logistic regression analysis and an optimized algorithm of the backward stepwise method conducted in the training set were employed to eliminate multicollinearity and select the best panel from these independent risk factors. Finally, the combination of AdA and Asp was defined as the ideal panel based on the training set (**Table 2**). The predictive nomogram showing the weights and points was displayed in **Figure 5A**. The area under the curve (AUC) of the ROC for the training set of recovered DILI vs. chronic DILI is 0.889, with a sensitivity of 73.7% and a specificity of 92.7%, while the AUC for the validation set is 0.888, with a sensitivity of 71.4% and a specificity of 91.3% (**Figure 5B**). We then evaluated the predictive ability of AdA and Asp (‘AdA-Asp model’) with other markers such as TB, DB, ALP and TCA in all the DILI patients, which has been reported to be correlated with DILI chronicity in previous studies (11, 12, 28). The AUC for TB, DB, ALP and TCA were 0.57, 0.55, 0.58, and 0.57, respectively, while AUC for the panel of AdA and Asp was 0.850 (**Figure 5C**). The calibration plot for the risk of chronicity showed an optimal agreement between the prediction by nomogram and actual observation (**Figure 5D**). In the decision curve analysis, the AdA-Asp model provided a considerable net benefit across the reasonable threshold range (**Figure 5E**), suggesting a promising clinical utility of this model.

**Table 2:** Final model of the multivariate logistic regression analysis.

Parameters	B	Wald	OR (95%CI)	P value
adrenic acid	0.285	11.09	1.33 (1.15-1.62)	0.0009
aspartic acid	0.069	6.98	1.07 (1.02-1.14)	0.0083

### Hepatic Transcriptome Revealed Suppressed Bile Acid Synthesis and Enhanced PUFA Metabolism With Lipid Peroxidation in DILI Patients

**Figure 6:**
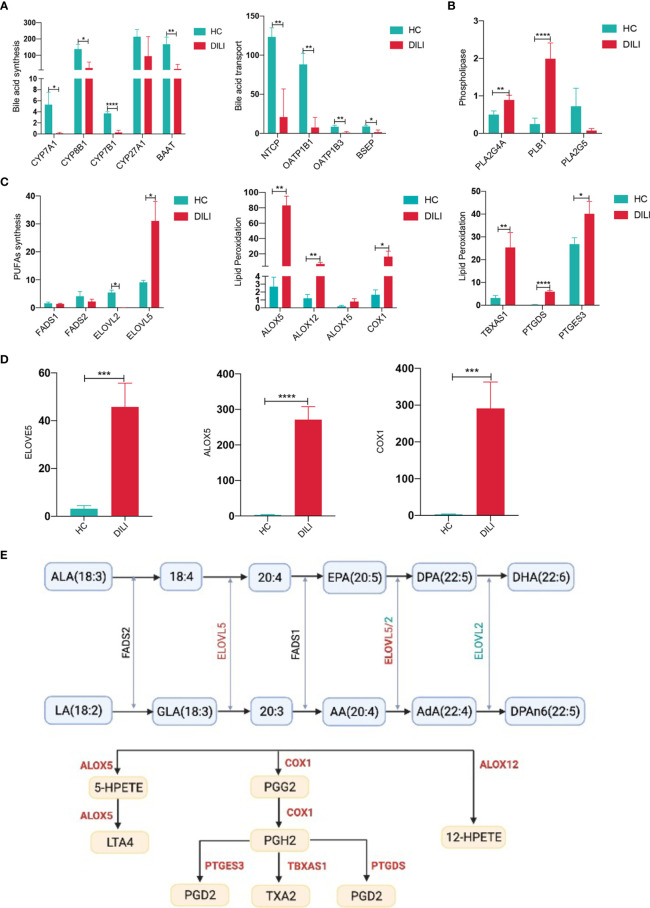
BA and PUFA metabolism related gene expression in liver of HCs and DILI patients. **(A)** Gene expression associated with bile acid synthesis in liver of HCs and DILI patients. **(B)** Altered phospholipase related gene expression in liver of DILI patients. **(C)** Gene expression associated with PUFA metabolism and lipid peroxidation in liver of HCs and DILI patients. **(D)** Verification of *ELOVE5*, *ALOX5* and *COX1* expression using qPCR. **(E)** A sketch map for the PUFA metabolism related enzymes (significant changes are shown as red for increase or green for decrease). *p < 0.05; **p < 0.01; ***p < 0.001; ****p < 0.0001. *NTCP*, sodium-BA cotransporter; *OATP1B*, organic anion transporting polypeptide 1B; *BSEP*, bile salt export pump; *CYP*, cytochrome P450; *PLA2*, phospholipaseA2; *PLB*, phospholipase B1; *FADS*, fatty acid desaturase; *ELOVL*, elongation of very long chain fatty acids protein; *ALOX*, arachidonic acid lipoxygenase; COX, cyclooxygenase; *TBXAS1*, thromboxane synthase 1; *PTGDS*, prostaglandin D2 synthase; *PTGES3*, prostaglandin E synthase 3, LTA4, leukotriene A4; TXA2, thromboxane A2; PGG2, prostaglandin G2; PGH2, prostaglandin H2; PGD2, prostaglandin D2.

The altered serum metabolites prompted us to examine the hepatic expression of genes related to BAs and PUFAs metabolism by PCR-array. As illustrated in **Figure 6A** and **SFigure 2A**, enzymes responsible for BA synthesis such as *CYP7A1*, *CYP8B1*, *CYP7B1* and *BAAT* showed a significant decrease in livers of DILI patients. Moreover, hepatic expression of genes involved in BA uptake, such as *NTCP*, *OATP1B1* and *OATP1B3* were decreased in DILI patients. Bile salt export pump (*BSEP)*, which pumps BAs into the bile canaliculus (29), also exhibited a reduced expression in DILI patients (**Figure 6A**). We then confirmed the decreased expression of *CYP7A1, NTCP*, *OATP1B1 and BSEP* using qPCR (**SFigure 2C**). Together with the alteration of BA transporters (**SFigure 2B**), the liver transcriptome revealed an impairment of BA synthesis and transportation in the liver of DILI patients.

Our previous data suggested the importance of PUFA metabolism in the chronicity of DILI, thus we further analyzed the expression of genes associated with PUFA metabolism. Phospholipase A2 group IVA (PLA2G4A) and phospholipase B1 (PLB1), which could phospholipase PUFAs and transfer PUFAs into nonestesterified fatty acid, were increased in liver of DILI patients (**Figure 6B**). Fatty acid desaturase 1 (*FADS1)* and fatty acid desaturase 2 (*FADS2)*, which are involved in the biosynthesis of AA from LA were comparable between DILI and HCs (**Figure 6C**). Besides, the expression of PUFA biosynthesis associated enzyme elongation of very long chain fatty acids protein 5 (*ELOVL5*) was observed to be significantly upregulated and *ELOVL2* was downregulated in DILI patients (**Figure 6C**). In addition, increased expression of genes responsible for PUFA peroxidation (30, 31), such as *ALOX5*, *ALOX12*, *COX-1*, *TBXAS1*, *PTGDS* and *PTGES3* were observed in liver of DILI patients (**Figure 6C**). We then confirmed the elevated expression of *ELOVL5*, *ALOX5* and *COX1* using qPCR (**Figure 6D**). A sketch map for the PUFA metabolism and related enzymes was shown in **Figure 6E**. The alteration of gene expression mentioned above suggested an increased peroxidation of PUFAs in DILI patients.

### Gut Microbiota Profile of DILI Patients and Its Correlation With BA and PUFA Metabolism

**Figure 7:**
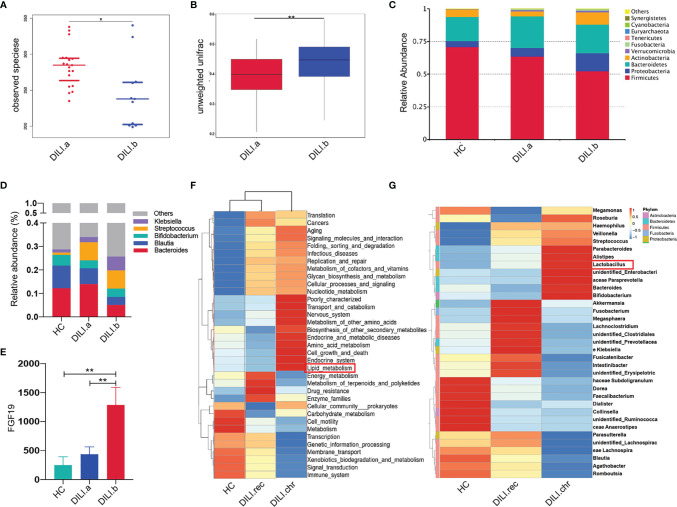
Gut microbiota profile of HCs and DILI patients. **(A)** The alpha diversity (observed species) index indicated the community diversity of DILI.a and DILI.b groups. **(B)** The beta diversity indices (unweighted wilcox) of the fecal microbiome in DILI.a and DILI.b group. **(C)** The relative abundance of different microbiota in HCs, DILI.a and DILI.b was shown in stacked bar chart (top 10 relevant abundance at phylum level). **(D)** The relative abundance of different microbiota in HCs, DILI.a and DILI.b was shown in stacked bar chart (top 5 relevant abundance at genus level). **(E)** Serum FGF19 levels in HC, DILI.a and DILI.b groups. **(F)** Cluster graph of gut microbiota function prediction using Tax4Fun (level 2). **(G)** Cluster graph of differential gut microbiome between HC, DILI.rec and DILI.chr at the genus level. *p < 0.05; **p < 0.01.

Given that gut microbiota is closely related to bile acid and PUFA metabolism (32, 33), 16S rRNA analysis was performed with stool samples from 30 DILI patients and 30 HCs. Analysis of alpha diversity showed that the richness and diversity of the gut microbiota in DILI patients were significantly decreased compared to HCs, especially in critical DILI patients (DILI.b group) (**SFigure 3A** and **Figure 7A**). Although the beta diversity was comparable between HCs and DILI (**SFigure 3B**), the microbial composition was distinct as evidenced by significantly different beta diversity when compared between non-critical (DILI.a) and critical (DILI.b) groups (**Figure 7B**).

At the phylum level, patients with DILI had lower levels of Firmicutes and Bacteroidetes, but higher levels of Proteobacteria and Actinobacteria compared with HCs (**Figure 7C**). These alterations were more obvious in critical DILI (DILI.b group) (**Figure 7C**). At the genus level, Bacteroides and Bifidobacterium were significantly decreased in DILI patients (**Figure 7D**). Linear discriminant analysis effect size (LEfSe) further showed that Clostridia and Clostridiales were significantly reduced in DILI patients compared with HCs (**SFigure 3C**).

As the BA receptor, farnesoid X receptor (FXR) in the intestine promotes the synthesis and release of fibroblast growth factor 19 (FGF19), which enters the portal venous circulation and suppresses BA synthesis in the liver (34, 35). To determine the function of gut microbiota in the regulation of BA metabolism, we further tested the levels of serum FGF19. The serum level of FGF19 in critical DILI patients (DILI.b group) was about 2 folds higher than that in non-critical DILI patients (DILI.a group) (p<0.01). (**Figure 7E**).

Furthermore, the difference of gut microbiota between the recovered and chronic group at the acute stage was also analyzed. The alpha diversity was comparable (**SFigure 4A**) while the beta diversity index indicated a difference of gut microbiota community composition between DILI.rec and DILI.chr group (**SFigure 4B**). Cluster analysis at the family level showed that lactic acid bacteria (LAB, including Enterococcaceae and Lactobacillaceae), responsible for the PUFA saturation (33, 36–38), were enriched in chronic DILI (DILI.chr) compared with recovered DILI (DILI.rec) or HC groups (**SFigure 4C**). At the genus level, the enrichment of Lactobacillus in chronic DILI was also observed (**Figure 7G**). Functional profiles of the microbiome were predicted using Tax4Fun (**SFigure 4D** **)**. As illustrated in **Figure 7F**, lipid metabolism was enhanced in the gut microbiota in chronic DILI patients. The function prediction using Tax4Fun revealed enhanced lipid metabolism in gut microbiota in chronic DILI patients (**SFigure 4C** and **Figure 7F**).

## Discussion

To our knowledge, this is the first study to systematically investigate the various serum metabolites to identify novel metabolic biomarkers for evaluating DILI progression and clinical prognosis. We found that serum levels of certain BAs were most associated with the severity of DILI, and provide the first evidence for omega-6 PUFAs as the potent markers for chronic DILI prediction.

Previous studies showed that BA metabolism was significantly altered in DILI patients and the disorders of BA homeostasis was one of the underlying mechanisms of DILI progression (11, 39, 40). GCA, TCA, glycodeoxycholic acid (GDCA), tauroursodeoxycholic acid (TUDCA), GCDCA, and taurodeoxycholate acid (TDCA) were associated with a high degree of liver damage in DILI (10, 11, 41). In our study, BA metabolism was found correlated with the severity of DILI and the levels of GCA, GCDCA, TCA and TCDCA were identified as potent biomarkers for evaluating the severity. Another promising finding in our study was that the BA converting related gut bacteria were decreased in the gut of patients with DILI. In the intestine, Bifidobacterium, Clostridia and Clostridiales are responsible for the transforming of primary BAs into secondary BAs *via* bile salt hydrolase (BSH), 7-alpha-dehydroxylase, or 7-alpha-hydroxysteroid dehydrogenase (7-alpha-HSDH) (29, 42), resulting in decreased production of secondary BAs such as UDCA, DCA and LCA. The reduced abundance of these species in DILI patients was in line with the imbalance of serum BAs. Our results suggested that the increased ratio of serum primary/secondary BAs might be due to decreased abundance of BA transforming bacteria in the gut of DILI patients.

Intestine FXR, regulated by BAs in the gut, could suppress the BA synthesis and transportation in the liver by the FGF19 pathway (34, 35). Secondary BAs especially UDCA activate FXR, whereas primary BAs especially CDCA inhibit FXR activation (43–47). Thus, we speculated that the decreased secondary BA production in the intestine might contribute to the enhanced intestine FXR activity in DILI patients. Activation of intestine FXR leads to the synthesis and release of FGF19, which could enter the portal venous circulation and inhibit BA synthesis in the liver (34, 35). Our speculation was further supported by the levels of increased serum FGF19 and decreased hepatic expression of *CYP7A1* and *CYP8B1* in DILI patients. Taken together, the present study demonstrated that alteration of gut microbiota might activate the intestine FXR-FGF19 signaling pathway and thus act as negative feedback to suppress BA accumulation in the liver of DILI patients.

Besides BAs, PUFAs were found to be significantly increased in DILI compared with HCs and we also revealed the relationship between serum PUFAs and the chronicity of DILI. PUFAs and their long-chain derivatives are important components of cell membranes. Increased oxidative stress is recognized as an underlying mechanism of DILI (39, 48), and oxidative stress can induce the activation of phospholipase (49, 50), resulting in dissociation and release of PUFAs from the cell membrane into circulation. In this study, the expression levels of phospholipase *PLA2G4A* and *PLB1* were found elevated in DILI patients, indicating that increased PUFAs in DILI might be due to upregulated phospholipase in liver which was induced by enhanced oxidative stress.

Further analysis showed that omega-6 PUFAs including GLA and AdA were closely correlated with the severity and chronicity of DILI. Accumulating evidence revealed that omega-6 PUFAs exert ‘bad’ effects on liver diseases, including induction of oxidative stress (51) and enhancement of inflammation (52). However, it is generally accepted that omega-3 PUFAs are mainly anti-inflammatory (24). The disorders of omega-6/3 PUFA metabolism were involved in the development of various liver diseases. For example, serum GLA is elevated in NAFLD and NASH (53), and the elevated ratio of omega-6/3 PUFAs in the diet increases the susceptibility to NAFLD (54). In the current study, serum omega-6 PUFAs including LA, GLA and AdA were found upregulated in chronic DILI patients. The ratio of (AdA+GLA+LA)/(EPA+DHA), which represents the ratio of omega-6/3, was significantly higher in DILI patients, especially in chronic DILI patients. These data suggested that the imbalance of omega-6 and omega-3 PUFAs was involved in the chronicity of DILI.

AdA and AA are important components of omega-6 PUFAs. The serum level of AdA was significantly upregulated in chronic DILI patients compared to recovered ones, however, the level of AA was comparable between the two groups. Interestingly, a similar alteration of AdA and AA has been reported in NAFLD and AdA has been reported to promote liver inflammation (51–53). The increased expression of *ELOVL5*, which transformed AA into AdA (55), suggested elevated synthesis of AdA. While the decreased expression of *ELOV2*, which was responsible for the biosynthesis of DPAn-6 from AdA (55), suggested the reduced consumption of AdA. These changes of gene expression might partly explain the alterations of AA and AdA in DILI.

Peroxidation of PUFAs could mediate inflammatory responses and enhance tissue injury, and was involved in the etiology of various liver diseases (56). It is found that oxidative fish oil could exacerbate the progression of alcoholic liver disease (57, 58). Among all kinds of PUFAs, AA and AdA are the most susceptible to lipid peroxidation (30). In our study, AA, AdA and (AdA+GLA+LA)/(EPA+DHA) were observed to be correlated with serum MDA and MDA/GSH-Px, which are indicators of lipid peroxidation. Notably, *ALOX5*, *ALOX12* and *COX-1*, which encode enzymes involved in lipid peroxidation (30, 31), were highly expressed in the liver of DILI patients, suggesting that increased products of PUFA peroxidation might lead to chronicity in DILI patients.

A further novel finding was that gut microbiota exhibited an altered function of lipid metabolism in chronic DILI. We observed enriched lactic acid bacteria, including Enterococcaceae, Lactobacillaceae at the family level and Lactobacillus at the genus level in the chronic DILI (DILI.chr) group, which were able to transform PUFAs into less toxic free saturated fatty acids serving as a detoxifying mechanism in the gut (36, 37). These results suggested that these enriched bacteria might reduce the toxicity of PUFAs, thus acting as a negative feedback mechanism to partially counteract the harmful effect of accumulated PUFAs in DILI patients. The presents data indicated that supplement of lactic acid bacteria might be a promising strategy for the prevention and treatment of chronic DILI.

Up till now, biomarkers for the early prediction of chronic DILI have not been defined. Metabolomic analysis can reveal the subtle changes of physiological and pathological status, thus has become a competitive strategy for exploring biomarkers in various diseases (59). In our study, serum metabolites were detected by using global metabolic analysis and logistic regression was used to select metabolites in order to establish the prediction model for chronic DILI. An ‘AdA-Asp model’ was developed in our study, showing better predictive ability than biochemical indicators such as TB, DB, ALP (28) and TCA (11).

By taking advantage of high-throughput experimental technologies, the present study shed new light on the importance of BAs and PUFAs in the progression of DILI. The predictive model developed in our study was based on a thorough examination of various metabolites and offered a simple and convenient strategy for the prediction of chronicity risk in DILI patients at an early stage, allowing early intervention and management for chronic DILI. However, our study is a single-center study with a limited number of patients. Further validation for the clinical practice of our Ada-Asp model is needed in a larger population.

Furthermore, our study suggested that at the acute stage of the disease, gut microbiota might exert a beneficial role in maintaining the homeostasis of BA and PUFA *via* an FGF19 signaling pathway and PUFA saturation respectively, although it seems that this negative feedback cannot totally counteract the accumulation of PUFAs and BAs. The function of gut microbiota in the regulation of BA and PUFA metabolism should be further confirmed by using germ-free mouse model or fecal microbiota transplantation. Further clinical studies are needed to explore the potency of gut microbiota modulation as a therapeutic strategy for DILI.

## Conclusion

In conclusion, BAs and PUFAs showed a strong association with the severity and chronicity of DILI. To be detailed, GCA, GCDCA, TCA and TCDCA could be potent markers for severity discrimination. The panel of AdA and Asp performed well in identifying DILI patients with a high risk of chronicity at the early stage of the disease, which could help with early intervention in clinical practice. Furthermore, we revealed the clue of gut microbiota in regulating BA and PUFA metabolism, which suggested that metabolites and gut microbiota might be new targets for DILI therapy. This study expanded our understanding of DILI progression and chronicity, and shed new light on optimizing the clinical management of DILI.

## Data Availability Statement

The original contributions presented in the study are included in the article/**Supplementary Material**. Further inquiries can be directed to the corresponding authors.

## Ethics Statement

The studies involving human participants were reviewed and approved by Ethics Committee of Shanghai Ruijin Hospital, School of Medicine, Shanghai Jiao Tong University. The patients/participants provided their written informed consent to participate in this study.

## Author Contributions

QX, SZ, XX, and XW contributed to conception and design of the study. SZ organized the database. SZ, HF, and TZ performed the statistical analysis. SZ wrote the first draft of the manuscript. MC, YH, QG, CZ, CQ, JW, and ZZ wrote sections of the manuscript. All authors contributed to manuscript revision, read, and approved the submitted version.

## Funding

This work was supported by the National Natural Science Foundation of China (No. 82070604, No. 81770587, No. 81970544, No. 81770578), Key Projects in the National Science & Technology Pillar Program during the Thirteenth Five-year Plan Period (2017ZX10203201-008, 2018ZX09201016-003-001, 2017ZX10202202-005-004), the Shanghai Municipal Key Clinical Specialty (shslczdzk01103) and the Shanghai RuijinHospital Three-Year Plan of the Clinical Skills and Innovations (2018CR005).

## Conflict of Interest

The authors declare that the research was conducted in the absence of any commercial or financial relationships that could be construed as a potential conflict of interest.

## Publisher’s Note

All claims expressed in this article are solely those of the authors and do not necessarily represent those of their affiliated organizations, or those of the publisher, the editors and the reviewers. Any product that may be evaluated in this article, or claim that may be made by its manufacturer, is not guaranteed or endorsed by the publisher.

## Glossary

**Table:**

7-alpha-HSDH	7-alpha-hydroxysteroid dehydrogenase
AA	arachidonic acid
AdA	adrenic acid
AIH	autoimmune hepatitis
ALA	α-linolenic acid
ALOX	arachidonic acid lipoxygenase
ALP	alkaline phosphatase
ALT	alanine aminotransferase
ANOVA	analysis of Variance
Asp	Aspartic acid
AST	aspartate aminotransferase
AUC	area Under Curve
BAAT	BA-CoA amino acid N-acyltransferase
BA	bile acid
BSEP	bile salt export pump
BSH	bile salt hydrolase
CA	cholic acid
CDCA	chenodeoxycholic Acid
CHB	chronic hepatitis B
COX	cyclooxygenase
CYP	cytochrome P450
DB	direct bilirubin
DCA	deoxycholic acid
DHA	docosahexaenoic acid
DILI	drug induced liver injury
DPA	docosapentaenoic acid
EDTA	ethylene diamine tetraacetic acid
ELOVL	elongation of very long chain fatty acids protein
EPA	eicosapentaenoic acid
FADS	fatty acid desaturase
FGF19	fibroblast growth factor 19
FXR	farnesoid X receptor
GCA	glycocholic acid
GCDCA	glycochenodeoxycholic acid
GDCA	glycodeoxycholic acid
GLA	gamma-linolenic acid
GSH-Px	glutathione peroxidase
HCs	healthy controls
HHT	hereditary hemorrhagic telangiectasia
KEGG	kyoto encyclopedia of genes and genomes
LA	linoleic acid
LAB	lactic acid bacteria
LCA	lithocholic acid
LEfSe	linear discriminant analysis effect size
LTA4	leukotriene A4
MDA	malondialdehyde
NAFLD	nonalcoholic fatty liver disease
NTCP	sodium-BA cotransporter
OATP1B1	organic anion transporting polypeptide 1B1
OATP1B3	organic anion transporting polypeptide 1B3
OTU	operational taxonomic unit
PCA	principal component analysis
PGD2	prostaglandin D2
PGG2	prostaglandin G2
PGH2	prostaglandin H2
PLA2G12A	phospholipase A2
group XIIA	PLA2G4A
phospholipase A2	group IVA
PLB1	phospholipase B1
PTGDS	prostaglandin D2 synthase
PTGES3	prostaglandin E synthase 3
PUFA	polyunsaturated fatty acid
RF	random forest
ROC	receiver operating characteristic
ROS	reactive oxygen species
SCFA	short chain fatty acids
SMPDB	small molecule pathway database
TB	total bilirubin
TBXAS1	thromboxane synthase 1
TCA	taurocholic acid
TCDCA	taurochenodeoxycholic acid
TDCA	taurodeoxycholate acid
TUDCA	tauroursodeoxycholic acid
TXA2	thromboxane A2
UDCA	ursodeoxycholic acid
GLA	gamma linolenic acid.
