# Supplementary material for: Alteration of Bile Acids and Omega-6 PUFAs Are Correlated With the Progression and Prognosis of Drug-Induced Liver Injury
Source: Front Immunol. 2022 Apr 12;13:772368. doi: 10.3389/fimmu.2022.772368 (PMC9041619; doi:10.3389/fimmu.2022.772368)
Supplement: Supplementary file 1 [file DataSheet_1.docx]

Supplementary Material

The Criteria of the healthy controls were as follows: (i) aged from 18 to 78 years; (ii) without drug taken within 2 weeks before blood collection; (iii) without a history of liver diseases, diabetes mellitus, hypertension, or malignancy; (iv)normal results on liver function, serum lipid, serum glucose, blood cell counts, renal function, B-abdominal ultrasound.

## Supplementary Figures

**SFig 1.** **Supplementary figures of metabolism analysis in DILI patients.**

(A) A pathway analysis used to discover the different metabolic characteristics between DILI.a and DILI.b (based on KEGG). (B) A pathway analysis used to discover the different metabolic characteristics between DILI.rec and DILI.chr (based on KEGG).


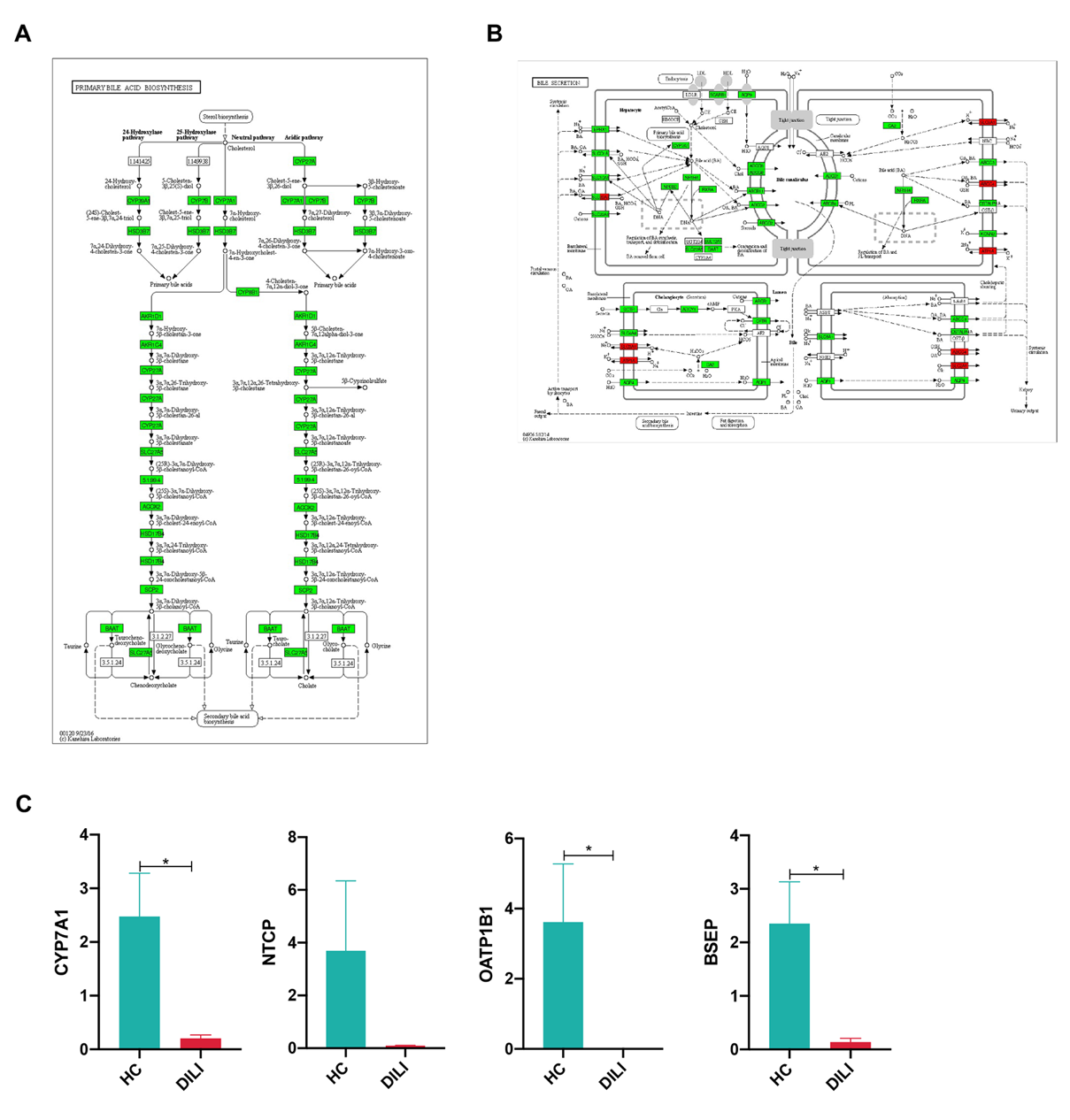


**SFig 2. Alteration of hepatic bile acid metabolism related gene expression in DILI patients.**

(A) Primary bile acid biosynthesis related genes expression in liver. (B) Bile acids secretion related genes expression in liver. Significant changes are shown as red for increase or green for decrease. (C) Verification of *CYP7A1,* *NTCP*, *OATP1B1*and *BSEP* expression using qPCR.

**
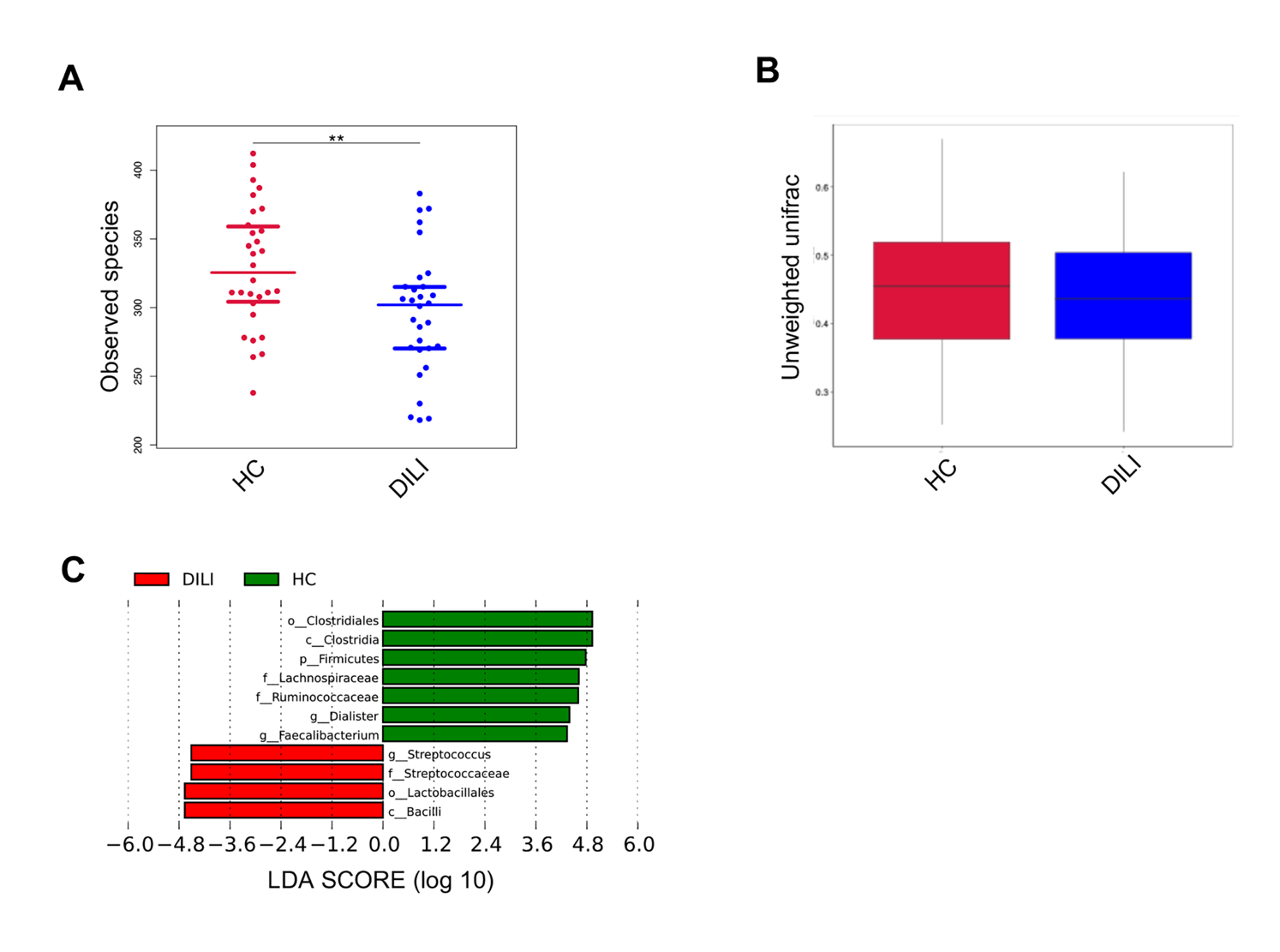
**

**SFig 3. Gut microbiota analysis between HCs and DILI patients.**

(A) The alpha diversity (observed species) index indicated the community diversity of DILI and HC group. (B) The beta diversity index in HCs and DILI patients. (C) LEfSe analysis revealed significant bacterial differences in fecal microbiota between DILI and healthy groups.

LEfSe, linear discriminant analysis effect size.


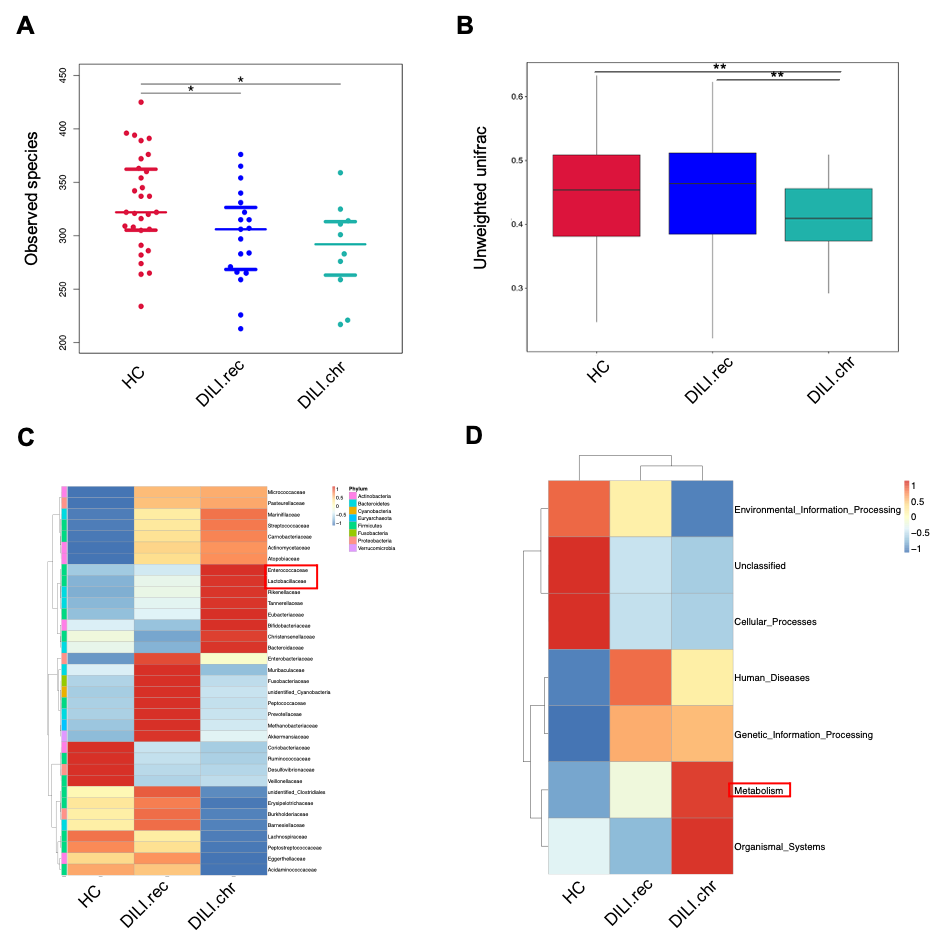


**SFig 4. Gut microbiota analysis between DILI.rec and DIIL.chr groups.**

(A) The alpha diversity (observed species) index in HC, DILI.rec and DILI.chr groups. (B) The beta diversity index in HC, DILI.rec and DILI.chr groups. (C) Cluster graph of differential gut microbiome between HC, DILI.rec and DILI.chr at the family level. (D) Cluster graph of function prediction using Tax4Fun (level 1).

## Supplementary Table

**STable 1. Detailed Drug Information in the DILI Cohort**

| **Parameters** | **Overall DILI**  n = 90 | **DILI.rec**  n = 65 | **DILI.chr**  n = 25 |
| --- | --- | --- | --- |
| **Herbs** | 60 (66.7%) | 43 (66.2%) | 17 (68.0%) |
| Polygonum multiflorum | 14 | 9 | 5 |
| Multiple herbs | 9 | 7 | 2 |
| Herbs for vasoactive | 5 | 4 | 1 |
| Herbs for arthralgia | 2 | 1 | 1 |
| Herbs for osteoporosis | 1 | 1 | 0 |
| Herbs for bronchiectasis | 1 | 1 | 0 |
| Notoginseng powder | 3 | 3 | 0 |
| Herbs for breast hyperplasia | 2 | 1 | 1 |
| Herbs for liver disease | 2 | 2 | 0 |
| Herbs for renal insufficiency | 4 | 3 | 1 |
| Herbs for common cold | 2 | 2 | 0 |
| Herbs for hyperthyroidism | 2 | 0 | 2 |
| Herbs for diabetes | 3 | 2 | 1 |
| Herbs for sjogren's syndrome | 1 | 0 | 1 |
| Herbs for edczema | 1 | 1 | 0 |
| Herbs for dyssomnias | 1 | 1 | 0 |
| Herbs for chronic pancreatitis | 1 | 0 | 1 |
| Herbs for cancer | 1 | 1 | 0 |
| Herbs for gastric polyps | 1 | 1 | 0 |
| Herbs for emphysema | 1 | 1 | 0 |
| Unknown herbs | 3 | 1 | 2 |
| **Conventional drugs** | 25 (27.8%) | 18 (27.7%) | 7 (28.0%) |
| Nonsteroidal anti-inflammatory drugs | 1 | 0 | 1 |
| Antibiotics | 1 | 1 | 0 |
| Statin | 3 | 2 | 1 |
| Antihyperthyroidism drug | 1 | 1 | 0 |
| Antihypertensive drugs | 2 | 1 | 1 |
| Immunomodulatory drug | 1 | 0 | 1 |
| Alpha receptor blocker | 1 | 1 | 0 |
| Proton pump inhibitors and serotonin receptor agonist | 1 | 1 | 0 |
| Immune checkpoint inhibitor | 7 | 6 | 1 |
| Anticoagulant drugs | 1 | 1 | 0 |
| Weight loss drugs | 1 | 1 | 0 |
| Nonsteroidal anti-inflammatory drugs and statin | 2 | 1 | 1 |
| Nonsteroidal anti-inflammatory drugs and antibiotics | 1 | 0 | 1 |
| Others | 2 | 2 | 0 |
| **Herbs and conventional drugs** | 5 | 5 | 0 |
| Fibrates and herbs for heart disease | 1 | 1 | 0 |
| Neurotransmitter drugs and herbs for Parkinson's Disease | 1 | 1 | 0 |
| Statin and herbs for vasoactive | 2 | 2 | 0 |
| Immune checkpoint inhibitor and herbs for cancer | 1 | 1 | 0 |

**STable 2. Univariate logistic regression analysis of metabolites and clinical characteristics**

| **Parameters** | **Estimate** | **Std. Erro** | **Z value** | **P value** |
| --- | --- | --- | --- | --- |
| GABA | -0.0109 | 0.0305 | -0.357 | 0.7208 |
| oxoadipic acid | -0.3105 | 0.7585 | -0.409 | 0.6822 |
| DPA | 1.2407 | 0.4797 | 2.586 | 0.0097 |
| heptanoic acid | -0.7011 | 1.3233 | -0.530 | 0.5960 |
| aspartic acid | 0.6034 | 0.2092 | 2.884 | 0.0039 |
| ornithine | 0.0120 | 0.0061 | 1.940 | 0.0524 |
| glyceric acid | -0.0110 | 0.0250 | -0.441 | 0.6595 |
| adrenic acid | 2.8774 | 0.8835 | 3.257 | 0.0011 |
| decanoic acid | 0.0006 | 0.0679 | 0.009 | 0.9927 |
| tyrosine | 0.0230 | 0.0081 | 2.834 | 0.0046 |
| GLA | 1.6093 | 0.5005 | 3.215 | 0.0013 |
| methylmalonic acid | 0.2635 | 0.1664 | 1.583 | 0.1134 |
| methionine | 0.0115 | 0.0068 | 1.704 | 0.0883 |
| malic acid | -0.0013 | 0.0041 | -0.317 | 0.7515 |
| lactic acid | 0.0007 | 0.0003 | 2.219 | 0.0265 |
| succinic acid | 0.3043 | 0.1407 | 2.162 | 0.0306 |
| hydroxyphenyllactic acid | 0.3945 | 0.1502 | 2.626 | 0.0086 |
| homovanillic acid | 0.5020 | 0.2956 | 1.698 | 0.0895 |
| fumaric acid | 1.8073 | 0.7004 | 2.580 | 0.0099 |
| TB | 0.0036 | 0.0024 | 1.523 | 0.1277 |
| DB | 0.0040 | 0.0045 | 0.905 | 0.3653 |
